# Point of Care Measurement of Body Mass Index and Thyroid Nodule Malignancy Risk Assessment

**DOI:** 10.3389/fendo.2022.824226

**Published:** 2022-02-11

**Authors:** Sara Ahmadi, Theodora Pappa, Alex S. Kang, Alexandra K. Coleman, Iñigo Landa, Ellen Marqusee, Matthew Kim, Trevor E. Angell, Erik K. Alexander

**Affiliations:** ^1^ Thyroid Section, Brigham and Women’s Hospital and Harvard Medical School, Boston, MA, United States; ^2^ Department of Medicine, Division of Endocrinology and Diabetes, Keck School Medicine of USC, Los Angeles, CA, United States

**Keywords:** BMI, obesity, thyroid nodule, thyroid cancer, nodule size

## Abstract

**Background:**

Large scale epidemiology studies have suggested obesity may increase the risk of thyroid cancer, though no prospective analyses using real-world measurement of BMI at a time proximate to initial thyroid nodule evaluation have been performed.

**Methods:**

We performed a prospective, cohort analysis over 3 years of consecutive patients presenting for thyroid nodule evaluation. We measured BMI proximate to the time of initial evaluation and correlated this with the final diagnosis of benign or malignant disease. We further correlated patient BMI with aggressivity of thyroid cancer, if detected.

**Results:**

Among 1,259 consecutive patients with clinically relevant nodules, 199(15%) were malignant. BMI averaged 28.6 kg/m^2^ (SD: 6.35, range:16.46-59.26). There was no correlation between the measurement of BMI and risk of thyroid cancer (p=0.58) as mean BMI was 28.9 kg/m^2^ and 28.6 kg/m^2^ in cancerous and benign cohorts, respectively. Similarly, BMI did not predict aggressive thyroid cancer (p=0.15). While overall nodule size was associated with increased BMI (p<0.01), these data require further validation as obesity may hinder nodule detection until large.

**Conclusion:**

In contrast to findings published from large scale association studies drawn from national databases, these prospective data of consecutive patients presenting for nodule evaluation detect no association of obesity (as measured by BMI) with thyroid cancer. Real time measurement of BMI at the time of thyroid nodule evaluation does not contribute to cancer risk assessment.

## Introduction

The detection of thyroid nodules has dramatically increased over the last thirty years, largely attributable to asymptomatic disease incidentally identified during physical examination or cross-sectional imaging. For most nodules larger than 1-1.5cm in diameter, identification leads to referral and subsequent fine needle aspiration (FNA) to rule out carcinoma. Not surprising given the expansion of evaluated nodules, the incidence of thyroid cancer has risen in parallel (1, 2).

Clinical and historical risk factors including childhood radiation exposure, familial thyroid cancer syndromes, and ultrasound features allow a healthcare provider to individualize thyroid nodule decision making at the point of care (3–6). Doing such can have profound impact upon reducing unnecessary intervention and cost, as patients participate in a shared decision-making process based on improved understanding of risk. Childhood exposure to ionizing radiation is perhaps the most robust historical factor increasing cancer risk (3, 4), yet is particularly infrequent among adult patients without a history of pediatric malignancy. Readily identifiable variables at the point of care such as age, sex, nodule size and nodule location have more recently been shown to impact cancer risk assessment in a nodule population (3, 7). Mathematical models have also been validated to allow for multivariate risk assessment and demonstrates how such variables can help modify cancer risk (3, 8).

Obesity, as measured by body mass index (BMI) may be another variable that can modulate thyroid cancer risk in a given patient and is readily measurable at the point of care. Indeed, several large scale epidemiology studies have suggested that obesity may increase the risk for thyroid cancer development (9–12), and also the aggressivity of thyroid cancer if detected (13–15). However, in contrast, other studies have reported no such association between obesity and thyroid cancer risk (16–18). One complexity in translating such data into clinical practice is to acknowledge that such large-scale association studies often carry selection or sampling bias. Few prospective analyses using real-world measurement of BMI at a time proximate to initial thyroid nodule evaluation have been performed. Such data would be invaluable in determining the value of including BMI in any multivariate risk assessment tool applicable at the point of care clinical assessment.

In the present prospective cohort analysis, we investigated patients presenting for initial nodule evaluation to better identidy the impact of BMI upon risk of thyroid malignancy using Brigham & Women’s Hospital thyroid nodule/cancer database. The Brigham & Women’s thyroid nodule clinic is the central referral center whereby all diagnostic evaluations of clinically relevant nodules occur within our healthcare system. This model of care substantially limits sample or selection bias while affording uniformity of clinical, radiologic and pathologic reporting.

## Methodology

We performed a 3-year, prospective cohort analysis investigating 1,360 consecutive patients presenting for initial evaluation of clinically significant thyroid nodules (>1cm in diameter) in our hospital system. Details of our thyroid nodule evaluation process have been described previously (3).

Demographic data such as age and sex were collected at the time of initial presentation, and we measured Body Mass Index (BMI) closest to time of clinic presentation and no greater than 6 months prior. BMI was calculated following medical assistant measurement of patient weight (obtained on a hospital clinic scale) and height (obtained using a hospital clinic stadiometer). If patients had multiple measurement of weight, the measurement closest to time of FNA was used.

We separately measured thyroid nodule size using 12-20mHz ultrasound, and ultimately sought to understand the impact of BMI upon the hard endpoint of benign or malignant disease. All patients underwent nodule evaluation in concordance with American Thyroid Association guideline recommendations (6). This included ultrasound evaluation, and ultrasound-guided fine needle aspiration (FNA). Non-diagnostic cytology led to a recommendation for repeat FNA in most circumstances, and nodules with indeterminate cytology were often further evaluated using Afirma molecular diagnostic tests applied to such scenarios. Benignity was confirmed when either a) cytology confirmed no malignant cells, b) histopathology confirmed benign findings, and/or c) negative molecular testing. Malignancy was confirmed only by histopathologic assessment following surgical resection, and the type of cancer was confirmed as well as documentation of typical pathologic features of disease (AJCC 8^th^ Edition).

To allow investigation into BMI’s hypothesized impact upon the aggressivity of thyroid cancer, we also divided all patients with thyroid cancer into two groups according to their histopathologic features. The higher risk thyroid cancer cohort included patients with anaplastic, medullary, and poorly differentiated carcinomas; any patient with distant metastatic disease; and those with Follicular/Hurthle cell carcinomas with vascular invasion. All others were defined as the lower risk thyroid cancer cohort.

We analyzed BMI as a continuous variable to determine overall impact on cancer risk. To further determine if extremes of BMI showed any signal of impact, we separately grouped all subjects into four defined weight categories according to their BMI [underweight (BMI ≤18.5 kg/m^2^), normal & overweight (BMI 18.6-29.9 kg/m^2^), obese (BMI 30-34.9 kg/m^2^) and morbidly obese (BMI ≥35 kg/m^2^)].

Statistical analysis was performed using the STATA/IC 16.1 statistical software. Logistic regression analysis was used to assess the effect of BMI as a continuous variable on the diagnosis of thyroid cancer. The Chi-Square test was used to assess the association between thyroid cancer and BMI quartiles. A T-test was used to assess the association between BMI and aggressiveness of thyroid cancer. We applied linear regression analysis to assess the association between BMI, largest nodule size, and nodule volume. P values <0.05 were considered significant. Permission for study was approved by the Brigham & Women’s Investigational Review Board (protocol # 1999P002899).

## Results

We enrolled 1,360 consecutive patients presenting for thyroid nodule evaluation over three years. Of these, 101 patients did not have a conclusive diagnosis or were lost to follow-up and were excluded. Thus, 1,259 patients defined our study cohort. Descriptive characteristics are shown in **Table 1**. The mean BMI of our cohort was 28.6 kg/m^2^ (SD 6.35, 16.46-59.26). Ultimately, 199 of 1259 patients (15.8%) were diagnosed with thyroid cancer.

**Table 1:** Patient and nodules characteristics.

**All patients: (n)**		1,259	
**Age (mean; SD)**		54.3 years (15)	
**Sex**		1,025 Female (81.4%)	
**Total nodules:**		2,494	
** Part of a Multinodular Goiter**		595 (48%)	
** Solitary nodules**		642 (52%)	
**Largest nodule size mean; SD**		2.4cm +/- 1.3cm	
**Patients with Malignant Nodule(s) (n=199)**		**Patients with Benign Nodule(s) (n=1,060)**	
Female (n)	146 (73.4%)	Female	879 (82.9%)
Age (mean; SD)	48.4 yrs (16.6)	Age	55.4 yrs (14.4)
BMI (mean, SD)	28.9 kg/m^2^ (6.8)	BMI (mean, SD)	28.6 kg/m^2^ (6.3)
**Type of Thyroid Cancer**			
**Papillary Carcinoma:**			
Classical Variant	100 (50.3%)		
Follicular Variant	55 (27.6%)		
Tall Cell Variant	11 (5.5%)		
Other PTC^1^	2 (1%)		
**Follicular Thyroid Carcinoma**	11(5.5%)		
**Medullary Thyroid Carcinoma**	3 (1.5%)		
**NIFTP**	11 (5.5%)		
**Poorly differentiated**	5 (2.5%)		
Anaplastic (arising in the background of TCVPTC)	1 (0.5%)		

NIFTP, Non-invasive Follicular Thyroid Neoplasm with Papillary-like Nuclear; TCVPTC, Tall cell Variant Papillary Thyroid Carcinoma.

**Figure 1:**
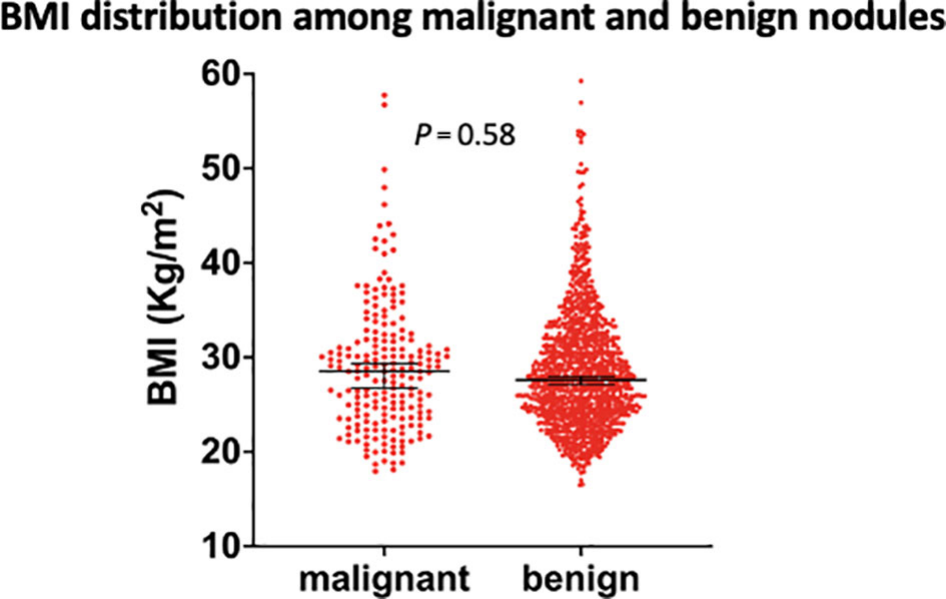
Comparison of body mass index levels between malignant and benign nodules. BMI, body mass index. Black vertical lines indicate mean and 95% confidence intervals.

The mean BMI among those with benign disease was 28.6kg/m^2^ compared to BMI 28.9 kg/m2 in those with thyroid cancer, and BMI did not correlate with benign or malignant disease (p=0.58, OR 1.007; 95% CI 0.98-1.03; **Figure 1**). This correlation remained non-statistically significant in multiple logistic regression analysis adjusting for age and gender (P=0.3).

To further assess any impact of BMI upon thyroid malignancy, we analyzed BMI specifically comparing those underweight (BMI ≤18.5) with those classified as morbidly obese (BMI ≥35) as these represent the extreme of a continuous spectrum. In total, 0.7% of our cohort was classified as underweight, while 14.3% was morbidly obese. Two of 11 underweight subjects harbored thyroid cancer, while 33 of 182 morbidly obese subjects harbored cancer, again confirming no association between thyroid cancer and extremes of BMI (p = 0.77).

We next investigated the influence of BMI upon aggressivity of disease among those harboring malignancy. Of the 199 patients with thyroid cancer, 14 (7%) were classified as high risk [3 (1.5%), 1 (0.5%), 5(2.5%) and 5 (2.5%) with medullary thyroid cancer, anaplastic thyroid cancer, follicular thyroid cancer with vascular invasion, and poorly differentiated thyroid cancer, respectively]. The average BMI was 26.4 kg/m^2^ among high-risk patients, while 29.1 kg/m^2^ among low-risk patients. There was no association between the aggressiveness of thyroid cancer and excess weight (p = 0.15). We also did not find any correlation between BMI and largest tumor size (P= 0.74).

**Figure 2:**
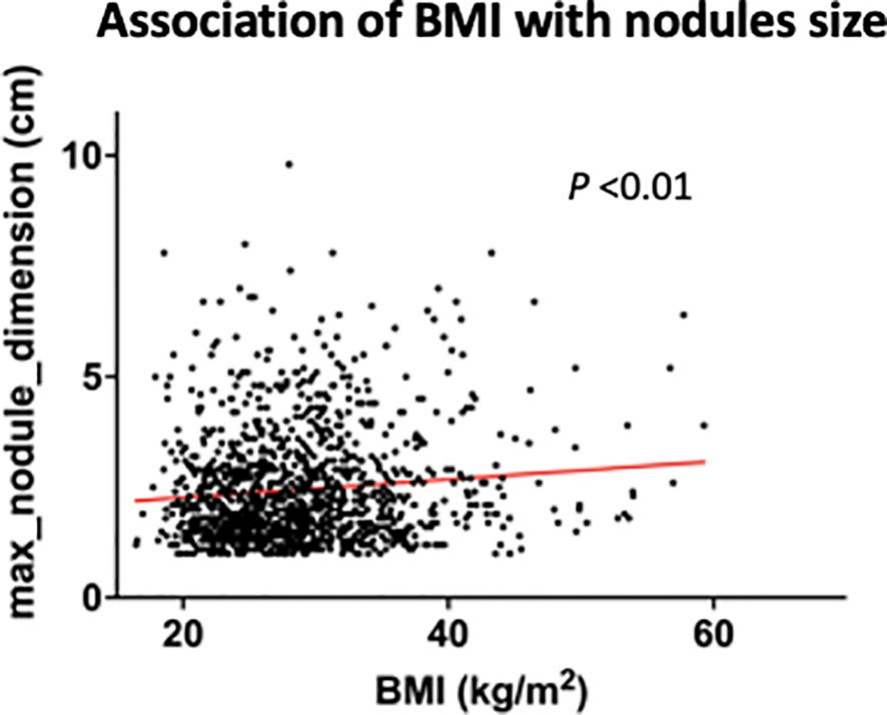
Correlation of body mass index with thyroid nodule size, noting that more obese patients harbored larger nodules (both benign & malignant). BMI, body mass index. Red line indicates regression line.

While no influence was detected upon malignant development or transformation of disease, we did identify an influence of BMI upon overall nodules dimension inclusive of both benign and malignant disease. Of the 1,259 patients, imprecise ultrasound measurement led to exclusion of 22, leaving 1,232 evaluable patients. The mean nodule dimension was 2.4 cm (SD=1.3cm), with an association between higher BMI and larger nodule diameter (P<0.01, Coef 0.02; **Figure 2**).

## Discussion

Several large association studies have suggested an influence of obesity upon thyroid tumorigenesis, though data are mixed and mechanistic hypotheses are uncertain (9–12, 15, 19–23). In a retrospective study of 351,402 subjects using Korean National Insurance System, Son et al. showed that risk of thyroid cancer was higher with increasing BMI in both men and women with a hazard ratio of 1.23 for obese and 1.26 for extremely obese patients (9). Similarly, Youssef et al. performed a meta-analysis of 31 studies reporting that weight gain increased the risk of developing thyroid cancer with Relative Risk (RR)=1.18, while weight loss decreased the risk with RR=0.89 (10). Another study from the National Institute of Health AARP-Diet and Health study cohort showed that overweight and obesity accounts for 13.6% of the annual percentage change in total PTC incidence rate and 57.8% of PTC > 4cm during a period 1995-2015 (12). However, an observational study in the UK biobank with benign thyroid nodules did not reveal an association between obesity and thyroid cancer (17), and this has been supported by others (18). In our prospective investigation we did not find an impact of obesity upon the cancer risk of any newly detected nodule. Most impactful to patient care is the ability to identify a risk variable proximal to the time of presentation such that individualized risk assessment can be applied to any decision-making process. Unfortunately, our data demonstrate that assessment of BMI at the point of initial thyroid nodule diagnostic evaluation is not clinically useful and would not significantly change individualized care decisions.

Obesity has been also investigated as a risk factor for aggressiveness of thyroid cancer (13, 14, 16, 24, 25). For example, Lee and colleagues retrospectively studied patients with PTC and showed strong associations between BMI with TNM stage (P<0.001) as well as BRAF V600E mutation status (P=0.008). They concluded that obesity can influence tumor progression in patients with thyroid cancer (13). In contrast, Matrone et al. in a study of patients with differentiated thyroid cancer patients failed to find any association between BMI and aggressiveness of thyroid cancer (16). Our prospective study similarly fails to confirm an association between obesity and developing more aggressive histology.

The application of findings from large database association studies to individualized care, is complex. At times, only through large multicenter registry studies can signals of association be detected, or enhanced understanding of diverse disease be elucidated. However, such large cooperative databases are prone to a lack of uniform processes for presentation or data entry, and even if association is detected, do not imply cause and effect. For these reasons, findings from such large association studies require further prospective validation especially to understand how novel findings are best applied to any individual clinical interaction. Though the broad impact of obesity upon thyroid cancer formation and neoplastic growth requires further investigation, our data suggests that point of care measurement of height and weight is not additive to individualized decision making regarding thyroid nodule care.

The strength of our data lies in the uniformity of data collection as well as nodule evaluation. BMI was routinely measured *via* hospital standards and by medical assistants trained in such procedures. Measurement tools for weight and height were standardized. A firm endpoint of benign or cancerous disease was confirmed with tissue-based analysis, either using cytology or histopathology, all by a single group of pathologists. Data on the population enrolled was collected prospectively and investigated consecutive patients over a three-year period.

We also acknowledge, however, limitations to our study. 101 patients were excluded from analysis due to lack of final diagnosis. However, it is highly likely that such a population of patients retains a mix of both benign and cancerous nodules and therefore would be unlikely to significantly impact our findings. Separately, a minority of patients had measurement of BMI a few months prior to their nodule FNA, and in theory could have lost (or gained) significant weight during the interim. Such scenarios, however, are rare on a population basis. Furthermore, short term impact of any weight loss upon a cancerous process is unlikely given the slow growth of nodular disease. One the other limitation of our study is the lack of data on BMI status over time. However, the importance of BMI status over time on risk of thyroid malignancy is unknown. We acknowledge that the Brigham & Women’s Hospital is a tertiary care center, and while the overall rate of malignancy is within comparable range to other publications, we acknowledge that slightly higher rates could be influenced by possible referral bias. If this were the case, our results may not translate to low-volume institutions or ones with a low overall incidence of malignancy.

Human randomized, controlled trials to investigate this clinical arena are impossible to perform, as randomization of a general population to become obese versus non-obese would be deemed unethical by most institutional review boards. Because of this, prospective, point of care, cohort analysis are powerful investigations. At a minimum, they inform us of which variable or finding will prove useful (or not) at the time of clinical decision making. Our findings can directly be applied to the clinical environment today, informing clinicians to not rely on weight or height for risk assessment.

In conclusion, real time assessment of BMI does not provide meaningful or actionable data for use in the clinical setting of thyroid nodule evaluation. Further studies should focus upon the possibility that obesity could influence overall nodule growth but must be designed to overcome a substantial sampling bias likely to occur in those presenting for nodule care.

## Data Availability Statement

The original contributions presented in the study are included in the article/supplementary material. Further inquiries can be directed to the corresponding author.

## Ethics Statement

This investigation was evaluated and approved by the Mass General Brigham Investigational Review Board.

## Author Contributions

All authors listed have made a substantial, direct, and intellectual contribution to the work, and approved it for publication.

## Conflict of Interest

The authors declare that the research was conducted in the absence of any commercial or financial relationships that could be construed as a potential conflict of interest.

## Publisher’s Note

All claims expressed in this article are solely those of the authors and do not necessarily represent those of their affiliated organizations, or those of the publisher, the editors and the reviewers. Any product that may be evaluated in this article, or claim that may be made by its manufacturer, is not guaranteed or endorsed by the publisher.
